# Managing Limbal Dermoids in Patients with Goldenhar Syndrome: A Case Series

**DOI:** 10.22336/rjo.2024.55

**Published:** 2024

**Authors:** Anchal Tripathi, Shalini Mohan, Lav Pathak

**Affiliations:** 1 Department of Ophthalmology, Military Hospital, Jammu, J&K, India; 2 Department of Ophthalmology, GSVM Medical College, Kanpur, UP, India; 3 ENT Department, Military Hospital, Jammu, J&K, India

**Keywords:** limbal dermoids, preauricular tags, cleft lip, Goldenhar syndrome, lamellar keratoplasty, simple excision, amniotic membrane transplant

## Abstract

**Objective:**

To investigate the clinical characteristics, associated systemic features, and management outcomes of patients with limbal dermoids diagnosed with Goldenhar syndrome.

**Methods:**

This case series included patients from the eye outpatient department, diagnosed with Goldenhar syndrome based on systemic evaluation. Demographic data, ophthalmological assessments, and systemic evaluations were recorded. Various surgical interventions were employed based on the extent of limbal dermoids, and postoperative follow-up spanned one year.

**Results:**

Seven patients (nine eyes) were included, with a mean age of 7.71±4.15 years. Limbal dermoids were bilateral in two patients and unilateral in five. Grade 1 and grade 2 limbal dermoids were observed. The mean BCVA was 0.50±0.11 (logMAR), and astigmatism was present in six patients. Systemic features of Goldenhar syndrome included vertebral anomalies, ear abnormalities, facial anomalies, and lower limb deformity. Surgical interventions varied, with simple excision, lamellar keratoplasty, and amniotic membrane transplantation utilized. Postoperatively, corneal scar formation occurred in all simple excision cases.

**Conclusion:**

This case series underscores the rarity of limbal dermoids in the context of Goldenhar syndrome and the importance of early surgical intervention in managing these cases. By sharing our experiences and outcomes, we hope to contribute to the broader understanding of this condition and its optimal treatment.

## Introduction

Goldenhar syndrome, also known as oculoauriculo-vertebral syndrome (OAVS), is a rare and complex congenital disorder characterized by a constellation of anomalies, including but not limited to limbal dermoids, pre-auricular tags, vertebral anomalies, cleft lip, and cleft palate, among others [1]. It is distinguished by the classic triad: oculo-auricular malformations, mandibular hypoplasia with facial asymmetry, and vertebral abnormalities [1,2]. Dr. Maurice Goldenhar first comprehensively described this enigmatic condition [2]. Goldenhar syndrome is exceptionally infrequent in the general population, being of special interest to clinicians and researchers alike.

The etiology of Goldenhar syndrome is multifactorial and, in part, presumed to have a genetic basis. While maternal factors such as diabetes, rubella infection, influenza infection, heavy alcohol consumption, and vitamin A intoxication have been implicated as potential contributors, the role of genetics remains paramount [3,4]. Additionally, certain medications, such as Thalidomide, retinoid acid, tamoxifen, and even cocaine intake during pregnancy, have been linked to the development of this syndrome [4,5]. As a result, Goldenhar syndrome represents a complex interplay between genetic and environmental factors.

Clinical diagnosis of Goldenhar syndrome poses a considerable challenge due to its variable and heterogeneous presentation [6]. Patients frequently first present to the Ophthalmology outpatient department with limbal dermoids, necessitating a high index of suspicion among healthcare providers to facilitate accurate diagnosis and appropriate management. Currently, no established diagnostic criteria for Goldenhar syndrome exist, to emphasize the pivotal role of clinical evaluation in confirming its presence. In this context, we present a case series of seven patients who came to our Ophthalmology outpatient department with limbal dermoids and were subsequently diagnosed with Goldenhar syndrome through comprehensive systemic evaluation.

## Materials and methods

This was a hospital-based case series study. All patients presenting to the eye outpatient department, from January 2022 to December 2022, with limbal dermoids, who were newly diagnosed with Goldenhar syndrome based on further systemic evaluation, were included in the study. Patients were excluded from the study in the following cases: guardians of the patients did not provide participation consent, no systemic association of limbal dermoids with Goldenhar syndrome was evident, and patients who sought medical attention for the recurrence of the condition and had previously received treatment elsewhere.

Written informed consent was obtained from the legal guardians of all participating children, ensuring compliance with the ethical standards of the Helsinki Declaration, and the Institutional Ethics Committee of our medical college approved the study.

All demographic data, including patient age, gender, and relevant medical history, were meticulously recorded. Comprehensive ophthalmological assessments, and systemic evaluations, were performed on each patient to identify any associated anomalies characteristic of Goldenhar syndrome. Cycloplegic retinoscopy was performed on all patients. Anterior segment optical coherence tomography (OCT) was performed on children who could sit unassisted, aiming to rule out anterior segment invasion. In cases where young patients could not cooperate, ultrasound biomicroscopy (UBM) was conducted under sedation. These tests were performed to rule out posterior tail extension of the dermoid.

The management of patients with Goldenhar syndrome adopted a multidisciplinary approach, acknowledging the varied nature of the condition. Regarding ophthalmological intervention, treatment modalities were determined based on the size and extent of the limbal dermoid. Options included simple excision, lamellar keratoplasty, or amniotic membrane transplantation. Preoperative and postoperative best-corrected visual acuity (BCVA) was noted. In addition to surgical interventions, postoperative refractive correction and occlusion therapy were administered to address any associated amblyopia.

Patients were diligently followed up for one year to monitor for any signs of recurrence or complications arising from the treatment.

## Results

We included nine eyes of seven patients. The mean age of the patients in our study was 7.71±4.15 years. Of the seven patients, three were females, and four were males. The guardians of the patients initially brought them to the Ophthalmology Department with the common complaint of progressively increasing growth in their eyes, which had been present since birth. This growth was accompanied by blurred vision and a foreign body sensation in the affected eye. The limbal dermoids were bilateral in two patients and unilateral in five patients. Grade 1 limbal dermoids (<5 millimeters) were found in three patients, while the rest of the patients (four) had grade 2 limbal dermoids, involving stroma and/or Descemet’s membrane [7].

**Fig. 1:**
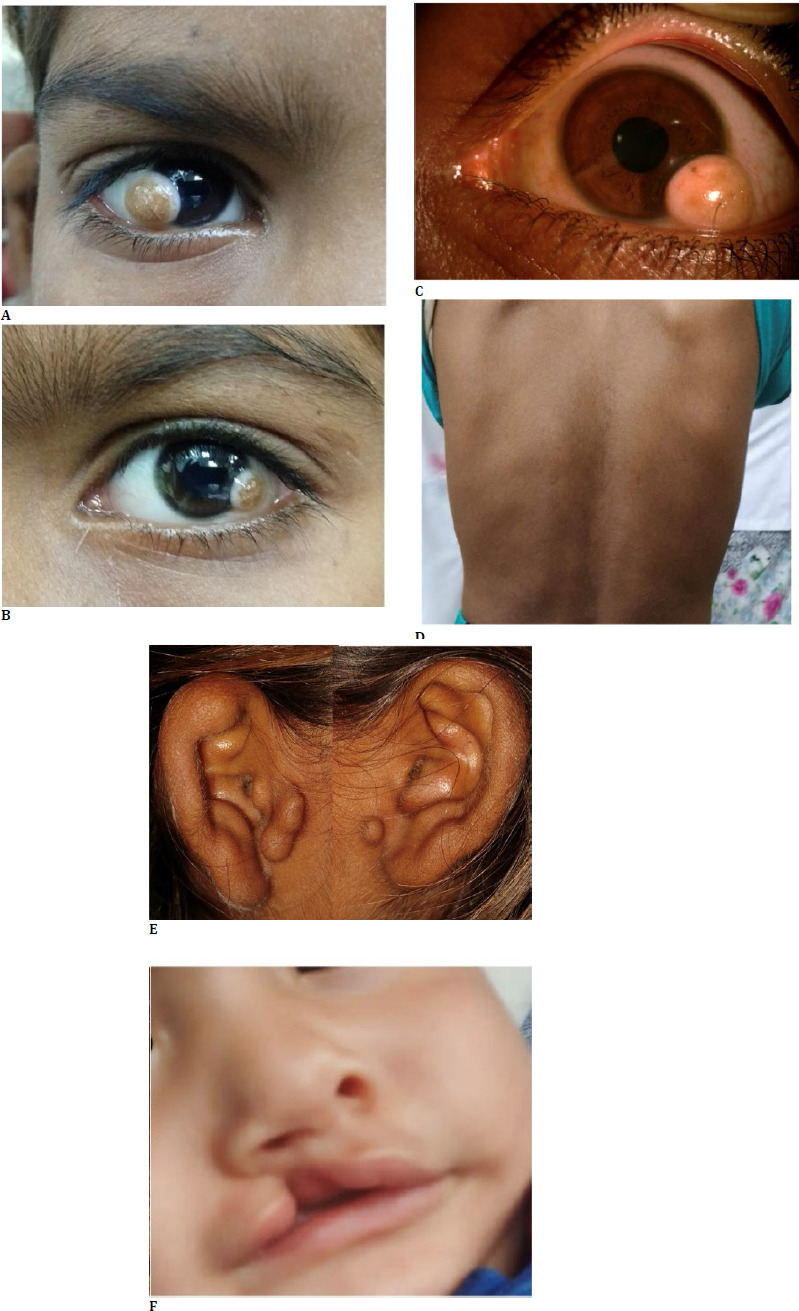
Oculo-auriculo-vertebral spectrum of Goldenhar syndrome; **A**. Grade two limbal dermoid; **B**. Grade one limbal dermoid; **C**. Grade two limbal dermoid with outgrowing hair follicle; **D**. Vertebral mild scoliosis; **E**. Pre-auricular tags; **F**. Cleft lip

The mean best-corrected visual acuity (BCVA) of the affected eye was 0.50±0.11 (logMAR). Six patients had astigmatism >1D, and one patient had astigmatism <1D. Notably, other ophthalmological findings were within normal limits. The association of additional features of Goldenhar syndrome in these patients is detailed in **Table 1**. Specifically, all patients exhibited vertebral anomalies, such as kyphosis, scoliosis, or both. Three patients had preauricular tags, while four patients presented with microtia. Additionally, five out of the seven patients displayed mandibular hypoplasia. One patient presented with a cleft lip, and another patient exhibited a lower limb deformity. **Fig. 1** shows the oculo-auriculo-vertebral spectrum of Goldenhar syndrome.

**Table 1:** Association of other systemic features of Goldenhar syndrome

S.No.	CLINICALFINDINGS	PATIENT1	PATIENT2	PATIENT3	PATIENT4	PATIENT5	PATIENT6	PATIENT7
**1**	**Eyeabnormalities**							
**(a)**	**Limbaldermoids**	+	+	+	+	+	+	+
**2**	**Earabnormalities**							
**(a)**	**Preauriculartags**	-	+	+	-	+	-	-
**(b)**	**Microtia**	+	-	-	+	-	+	+
**3**	**Vertebralanomalies**							
**(a)**	**Kyphosis**	+	+	-	-	+	+	-
**(b)**	**Scoliosis**	+	-	+	+	+	+	+
**4**	**Facialanomalies**							
**(a)**	**Mandibularhypoplasia**	-	+	-	+	+	+	+
**(b)**	**Cleftlip**	+	-	-	-	-	-	-
**5**	**Lowerlimbdeformity**	-	-	-	-	+	-	-

**Fig. 2:**
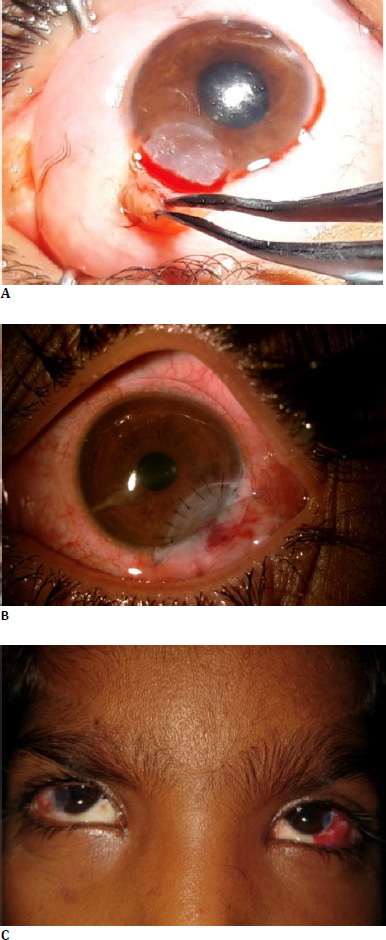
Tailored surgical management options for limbal dermoids; **A**. Simple excision; **B**. Sectoral lamellar keratoplasty; **C**. Sectoral lamellar keratoplasty in the right eye and small inlay amniotic membrane graft in the left eye of the same patient

For the management of limbal dermoids, simple excision was deemed sufficient for two patients (**Fig. 2 A**). Three patients required lamellar keratoplasty (**Fig. 2 B, C**), and in two patients, an inlay amniotic membrane transplant was performed (**Fig. 2 C**). **Table 2** shows a comparison of different surgical procedures in the management of limbal dermoids. Cycloplegic refraction was carried out four weeks post-operatively. Three patients had remaining amblyopia, for which occlusion therapy was advised.

**Table 2:** Comparison of different surgical procedures in the management of limbal dermoids

S.No.	PROCEDURE	MEAN AGE (years) (Mean±SD)	SIZE(mm) (Mean±SD)	PREOPERATIVE MEAN BCVA (logMAR) (Mean±SD)	POSTOPERATIVE MEAN BCVA (logMAR) (Mean±SD)	ASTIGMATISM (Diopters) (%)	SEQUELAE(%)
**1**	**SIMPLEEXCISION**	3.0±2.83	4.25±0.35	0.7±1.2	0.4±0.3	50%-<1D 50%- >1D	Cornealscarring at excision site- 100%
**2**	**SECTORALLAMELLAR KERATOPLASTY**	8.0±2.65	5.33±1.53	0.8±1.4	0.5±0.31	>1D	Amblyopia-75%
**3**	**AMNIOTICMEMBRANE TRANSPLANT**	11.5±0.71	6.33±1.15	1.0±0.71	0.7±0.62	>1D	Amblyopia-50%

The intraoperative and postoperative periods were uneventful in all the cases. Corneal scar formation was observed in all the patients who underwent simple excision, after a mean duration of 6.6 months post-operative.

## Discussion

The elucidation of limbal dermoids in our hospital-based case series has provided novel scientific insights, enriching our understanding of this rare ocular anomaly. Limbal dermoid processes, though relatively uncommon, have a notable global prevalence estimated at 1-3 cases per 10,000 individuals [1,8]. Strikingly, they exhibit no gender bias or racial predisposition [8,9]. While limbal dermoids are typically not hereditary, exceptions exist, particularly when they co-occur with systemic conditions like Goldenhar syndrome [8,9]. In such familial cases, the inheritance pattern often follows a multifactorial model [8,9].

Notably, limbal dermoids often manifest alongside additional ocular abnormalities. These can encompass eyelid colobomas, lacrimal gland irregularities, and staphyloma, affecting both the sclera and cornea [6,8,9]. Choristomas, which entail tissues’ or cells’ presence in atypical locations, frequently coexist with systemic diseases and syndromes like Goldenhar syndrome [8,9]. These associated features could include preauricular fistulas, preauricular appendages, ocular epithelioid or lipodermoid growths, and even mandibular dysostosis linked to Franceschetti syndrome [8-13].

Limbal dermoids are congenital anomalies, that emerge at birth and demonstrate an inclination to enlarge over time. An anatomical classification discerns three grades, serving as a foundational framework for clinical and surgical management: Grade I Limbal Dermoids are superficial lesions, usually measuring less than 5 mm in size, positioned at the limbus [7]. They may contribute to the development of anisometropic amblyopia, characterized by gradual progression, oblique astigmatism, and adjacent corneal flattening [7]. Grade II Limbal Dermoids encompass larger lesions that extensively cover the cornea, penetrating deep into the stroma and occasionally reaching Descemet’s membrane without involving the cornea [7]. While less common, grade III dermoids are the most extensive, completely enveloping the cornea and extending through the tissue structures spanning from the anterior eye surface to the pigmented epithelium of the iris [7]. The anatomical classification employed in our study, a fundamental aspect of our methodology, facilitated a nuanced severity assessment and guided our tailored treatment strategies. The predominance of Grade II limbal dermoids in our series, characterized by extensive corneal penetration, underscores the importance of a meticulous approach to surgical interventions, considering the depth and extent of these lesions.

Treatment options for limbal dermoids include simple excision coupled with superficial keratotomy, a conventional approach [6,7,14]. Nevertheless, this method carries inherent risks, such as corneal opacification, neovascularization, and the development of pseudopterygium [6,7,14]. Alternatively, surgeons may opt for lamellar keratoplasty post-lesion removal, offering the advantage of mitigating postoperative complications [6,7,14].

Although graft failure due to graft rejection is rare in lamellar keratoplasty, compared to simple excision, mastering this technique demands surgical expertise of a higher level [6,7,14]. The precision in limbus removal without Descemet’s membrane perforation represents the chief challenge [6,7,14]. Furthermore, the meticulous preparation of lamellar corneal grafts of consistent thickness is pivotal for favorable outcomes [6,7,14].

In summary, while lamellar keratoplasty presents greater surgical demands, it is an advantageous choice in limbal dermoids management, notably in minimizing post-operative complications and optimizing long-term results. Our study’s contribution to the treatment modalities’ understanding resonates with existing literature, highlighting the delicate balance between simple excision with superficial keratotomy and the sophisticated, yet advantageous, approach of lamellar keratoplasty. The meticulous one-year follow-up in our cases yielded valuable insights into postoperative outcomes, specifically corneal scar formation following simple excision, thus emphasizing the necessity for prolonged monitoring.

## Conclusion

Our hospital-based case series elevates the scientific discourse surrounding limbal dermoids, providing nuanced insights into their clinical characteristics, associations with Goldenhar syndrome, and the efficacy of tailored treatment approaches. The findings underscore the imperative of continued scientific inquiry to refine management strategies and unravel the underlying genetic and molecular complexities associated with this enigmatic ocular anomaly.
